# A case-control study on the risk factors for hemorrhagic fever with renal syndrome

**DOI:** 10.1186/s12879-020-4830-5

**Published:** 2020-02-04

**Authors:** Xiaochen Wang, Wenqi Shen, Yuanfang Qin, Liang Ying, Haipeng Li, Jiankui Lu, Jing Lu, Nan Zhang, Zhifeng Li, Weizhong Zhou, Fenyang Tang, Fengcai Zhu, Jianli Hu, Changjun Bao

**Affiliations:** 1 Department of Acute Infectious Disease Control and Prevention, Jiangsu Provincial Center for Disease Control and Prevention, Nanjing, 210009 China; 2 Department of Acute Infectious Disease Control and Prevention, Lianyungang Municipal Center for Disease Control and Prevention, Lianyungang, 222002 China; 3 Department of Acute Infectious Disease Control and Prevention, Guanyun County Center for Disease Control and Prevention, Lianyungang, 222002 China; 4 Department of Acute Infectious Disease Control and Prevention, Haizhou County Center for Disease Control and Prevention, Lianyungang, 222002 China

**Keywords:** Hemorrhagic fever with renal syndrome, HFRS, Case-control study, Community-based

## Abstract

**Background:**

Hemorrhagic fever with renal syndrome (HFRS) is an endemic communicable disease in China, accounting for 90% of total reported cases worldwide. In this study, the authors want to investigate the risk factors for HFRS in recent years to provide the prevention and control advices.

**Methods:**

A community-based, 1:2 matched case-control study was carried out to investigate the risk factors for HFRS. Cases were defined as laboratory-confirmed cases that tested positive for hantavirus-specific IgM antibodies. Two neighbourhood controls of each case were selected by sex, age and occupation. Standardized questionnaires were used to collect information and identify the risk factors for HFRS.

**Results:**

Eighty-six matched pairs were investigated in the study. The median age of the cases was 55.0 years, 72.09% were male, and 73.26% were farmers. In the multivariate logistic regression analysis, cleaning spare room at home (OR = 3.310, 95%CI 1.335–8.210) was found to be risk factor for infection; storing food and crops properly (OR = 0.279 95%CI 0.097–0.804) provided protection from infection.

**Conclusion:**

Storing food and crops properly seemed to be protective factor, which was important for HFRS prevention and control. More attention should be paid to promote comprehensive health education and behaviour change among high-risk populations in the HFRS endemic area.

## Background

Hemorrhagic fever with renal syndrome (HFRS), formerly known as epidemic hemorrhagic fever (EHF), is caused by several rodent-borne hantaviruses in the Hantaviridae family of the order Bunyavirales [1, 2]. HFRS is clinically characterized by fever, haemorrhage, kidney disease, headache, back pain, abdominal pain and hypotension [3–5]. Every year, thousands of people are infected by hantaviruses in developing and developed countries alike, case fatality rates range from 0.5 to 40% depending on the viral strain [2, 6, 7]. HFRS cases have occurred mainly in China, the Republic of Korea, and the Far East region of the Russian Federation in Asia, as well as in Finland, Sweden and western and central Europe [4, 8]. Approximately 150,000 to 200,000 patients with HFRS are hospitalized each year throughout the world [8]. With the highest occurrence of HFRS, China has experienced approximately 90% of the cases worldwide over the last few decades [3–5, 9]. From 2008 to 2018, a total of 118,124 cases of HFRS were reported in the country, making HFRS a critical public health issue. Transmission of hantaviruses is thought to occur mainly through contact with infected animal excreta or ingest food contaminated with infected animal excreta (faeces, urine and saliva). Though the aerosol route of infection is undoubtedly the most common means of transmission among rodents and to humans [5, 8, 10–12], virus transmission by bite occurs among rodents and may also result in human infection [8].

Only a few literatures analyzed the risk factors of HFRS in China in the past decade, especially in Jiangsu Province, only one paper reported in 2003 [13]. With the improvement of people’s living standards, the risk factors of morbidity may also change. In order to understand the possible risk factors of HFRS in Jiangsu Province in recent years, a community-based case-control study was conducted in our study to identify the risk factors for HFRS and to supplement the previous studies.

## Methods

### Case and control definition

The cases in this study were defined as confirmed cases that had disease onset during October 2015 and December 2016. Serum samples from suspected patients and identified matched controls were collected from Lianyungang City of Jiangsu Province. The diagnosis of suspected cases and confirmed cases were based on the Diagnosis Criteria for Epidemic Hemorrhagic Fever (WS278–2008) issued by the former Ministry of Health, People’s Republic of China as described elsewhere [14, 15]. Confirmed HFRS patients were defined as persons who present with specific IgM antibodies against hantavirus. The eligible controls were defined as neighbors of the case subjects who were matched by age (within 5 years), sex, occupation (farmer or non-farmer), length of local residence (longer than 6 months), and who were negative for specific IgG antibodies and without HFRS vaccine history. The controls were chose among the people who lived in the same community or village but at least 3 households (about 100 m) apart from the case subjects.

### Microbiological analyses

Serum samples from suspected patients and identified matched controls were collected and transported in a cold box (4–8 °C) to JSCDC’s laboratories for testing. Hantavirus-specific IgM antibodies were examined with the Colloidal Gold Diagnostic Kit for Antibody to Hanta virus (Boson Biotech, Xiamen, China) according to the manufacturer’s instructions.

### Data and samples collection

Eighty-six confirmed HFRS patients were selected as cases and 172 controls were recruited following the 1:2 matching method. Though occurred in every month, HFRS in Lianyungang had obvious seasonal character, reaching the peaks in autumn and winter, which generally started from October to February. Therefore, the investigation time was set to be from October 2015 to the whole year 2016, to ensure that the the epidemic period could be included, and extended appropriately to ensure the sample size.

A standardized questionnaire was used to investigate both cases and controls. Trained investigators interviewed the study subjects about their demographics (age, gender), living and working environment (e.g. presence of rats, pond, frequency of airing quilts), exposure history within 1 month prior to disease onset (e.g. raising live stocks or poultry, contacting with rats and/or their excreta, resting on grass field, broken skins, mite bites) and other possible risk factors (e.g. having underlying diseases, annual household income, taking rodent control measures). The questionnaires of cases were completed within 2 weeks after laboratory diagnosis. All questionnaires were systematically verified by JSCDC’s research coordinators for data integrity.

### Statistical analyses

Data was double entered into Epidata 3.1 (the Epidata Association, Denmark, Europe), and a database consistency check was performed. SPSS version 18.0 (the Statistical Product and Service Solutions, Chicago, IL, USA) was used for all statistical analysis. Univariate and multivariate logistic regressions were used to identify the risk factors for HFRS. The odds ratios (ORs) with 95% confidence intervals (CIs) were used to quantify the association strength between variables. Variables with *P* ≤ 0.10 in the univariate analysis were included in the multivariate regression model. The backward stepwise elimination procedure was applied to exclude the variables with *P* > 0.05 in the multivariate regression model.

## Results

This study had 258 participants, including 86 cases and 172 matched controls. For the cases and controls, the median ages were 55 years and 56.5 years; males accounted for 72.1 and 69.8%, 73.3 and 68.6% were farmers respectively (Table 1). There were no significant differences in demographic characteristics between cases and controls.

**Table 1:** Demographic matching factors of the cases and controls

Matching factors	Cases (*n* = 86)	Controls (*n* = 172)	*P*-value
	n(%) or mean ± SD	n(%) or mean ± SD	
Age	55.0 ± 15.2	56.5 ± 14.5	0.506
Sex			
Male	62 (72.1)	120 (69.8)	0.699
Female	24 (27.9)	52 (30.2)	
Occupation			
Farmer	63 (73.3)	118 (68.6)	0.442
Non-farmer	23 (26.7)	54 (31.4)	

*SD* Standard deviation

In univariate conditional logistic regression model, cleaning spare rooms at home, presence of rats at home were associated with a higher risk of HFRS; the following factors were associated with a reduced risk of being infected: household income> 30,000 RMB, taking rodent control measures at home, taking rodent control measures in the working areas, raising cats, dogs, chickens and ducks, airing quilts frequently, proper storage of food and crops and heating leftovers before eating. Other factors were not significantly different between cases and controls (Table 2).

**Table 2:** Univariate logistic regression analyses of potential risk factors

Variable	Cases	Controls	*P*-value	OR (95% CI)
	n(%)	n(%)		
Education level				
Primary school and below	51 (59.3)	86 (50.0)	0.101	0.595 (0.320–0.108)
Above the primary school	35 (40.7)	86 (50.0)		
Having underlying diseases				
Yes	5 (5.8)	13 (7.6)	0.585	0.733 (0.240–2.239)
No	81 (94.2)	159 (92.4)		
Household income (RMB)				
≤30,000	53 (61.6)	81 (47.1)	0.009	0.413 (0.212–0.804)
> 30,000	33 (38.4)	91 (52.9)		
House near pond				
Yes	38 (44.2)	70 (40.7)	0.533	1.264 (0.606–2.635)
No	48 (55.8)	102 (59.3)		
Cleaning spare rooms at home				
Yes	62 (72.1)	90 (52.3)	0.001	3.280 (1.653–6.512)
No	24 (27.9)	82 (47.7)		
Sprinkling while cleaning spare rooms at home				
Yes	9 (25.7)	37 (54.4)	0.418	0.549 (0.128–2.344)
No	26 (74.3)	31 (45.6)		
Presence of rats at home				
Yes	50 (58.1)	76 (44.2)	0.013	2.278 (1.188–4.366)
No	36 (41.9)	96 (55.8)		
Rodent control measures at home				
Yes	26 (30.2)	73 (42.4)	0.028	0.472 (0.242–0.921)
No	60 (69.8)	99 (57.6)		
Presence of rats in the working areas				
Yes	19 (22.1)	35 (20.3)	0.703	1.156 (0.548–2.438)
No	67 (77.9)	137 (79.7)		
Rodent control measures in the working areas				
Yes	2 (2.3)	25 (14.5)	0.008	0.064 (0.008–0.492)
No	84 (97.7)	147 (85.5)		
Contacting with rats and/or their excreta				
Yes	8 (9.3)	18 (10.5)	0.752	0.858 (0.332–2.216)
No	78 (90.7)	154 (89.5)		
Raising cats				
Yes	12 (14.0)	46 (26.7)	0.011	0.378 (0.179–0.797)
No	74 (86.0)	126 (73.3)		
Raising dogs				
Yes	30 (34.9)	80 (46.5)	0.054	0.562 (0.313–1.011)
No	56 (65.1)	92 (53.5)		
Raising chickens				
Yes	32 (37.2)	83 (48.3)	0.041	0.511 (0.268–0.973)
No	54 (62.8)	89 (51.7)		
Raising ducks				
Yes	5 (5.8)	26 (15.1)	0.012	0.143 (0.031–0.654)
No	81 (94.2)	146 (84.9)		
Raising gooses				
Yes	4 (4.7)	3 (1.7)	0.199	2.667 (0.597–11.975)
No	82 (95.3)	169 (98.3)		
Airing quilts				
Frequent(≥4 times per week)	59 (68.6)	142 (82.6)	0.004	0.328 (0.155–0.694)
Infrequent(< 4 times per week)	27 (31.4)	30 (17.4)		
Proper storage of food and crops				
Yes	24 (27.9)	67 (39.0)	0.028	0.442 (0.214–0.916)
No	62 (72.1)	105 (61.0)		
Heat leftovers before eating				
Yes	64 (74.4)	111 (64.5)	0.054	1.975 (0.987–3.951)
No	22 (25.6)	61 (35.5)		
Resting on a grass field				
Yes	7 (8.1)	6 (3.5)	0.066	4.464 (0.906–23.821)
No	79 (91.9)	166 (96.5)		
Farming with broken skins				
Yes	2 (2.3)	8 (4.7)	0.381	0.500 (0.106–2.355)
No	84 (97.7)	164 (95.3)		
Mite bites				
Yes	3 (3.5)	6 (3.5)	1.000	1.000 (0.250–3.998)
No	83 (96.5)	166 (96.5)		

All exposure factors were within the previous 1 month prior to disease onset

*OR* Odds ratio, *CI* Confidence interval

Luo et al. [16] reported in 1985 that both cats and rodents might be related to the spread of HFRS, while cat owners were more likely to develop HFRS, probably because the rodents infestation were more serious in the cat owners’ home. Meanwhile, dogs had also been reported to be naturally infected with HFRS virus [13], thus stratified analyses were conducted in this study to explore the individual and joint effects of raising cats and presence of rats at home, as well as the individual and joint effects of raising cats and raising dogs. All pairs were divided into case group and control group, with sex (*p* > 0.05) and age (*p* > 0.05) comparable between groups.

The results showed that it was more likely for participants with rats present at home to develop HFRS (*p* < 0.05). No significant results were found both in the individual effect of raising cats (*p* > 0.05) and in the joint effect of raising cats and rats present at home (*p* > 0.05) (Table 3). Additionally, there was no significant difference neither in the individual effect nor in the joint effect of raising cats and raising dogs (*p* > 0.05) (Table 4).

**Table 3:** Logistic regression analysis of interaction between presence of rats at home and raising cats

Exposure factors	Regression coefficient	Regression coefficient standard error	*P*-value	OR (95% CI)
Presence of rats at home	0.724	0.338	0.032	2.063 (1.065–3.998)
Raising cats	−0.216	0.579	0.710	0.806 (0.259–2.508)
Presence of rats at home by Raising cats	−0.814	0.769	0.290	0.663 (0.126–3.482)

All exposure factors were within the previous 1 month prior to disease onset

*OR* Odds ratio, *CI* Confidence interval

**Table 4:** Logistic regression analysis of interaction between raising cats and raising dogs

Exposure factors	Regression coefficient	Regression coefficient standard error	*P*-value	OR (95% CI)
Raising cats	−0.387	0.712	0.587	0.679 (0.168–2.742)
Raising dogs	−0.209	0.321	0.515	0.812 (0.433–1.522)
Raising cats by Raising dogs	−0.411	0.846	0.628	0.663 (0.126–3.482)

All exposure factors were within the previous 1 month prior to disease onset

*OR* Odds ratio, *CI* Confidence interval

In multivariate conditional logistic regression model, one variable was a significant risk factor for HFRS: cleaning spare room at home (OR = 3.310, 95%CI 1.335–8.210); one variables represented significant protective factor for HFRS: storing food and crops properly (OR = 0.279 95%CI 0.097–0.804) (Table 5).

**Table 5:** Multivariate logistic regression analyses of potential risk factors

Exposure factors	Partial regression coefficient	Partial regression coefficient standard error	*P*-value	OR (95% CI)
Cleaning spare rooms at home	1.197	0.463	0.010	3.310 (1.335–8.210)
Proper storage of crops	−1.276	0.540	0.018	0.279 (0.097–0.804)

All exposure factors were within the previous 1 month prior to disease onset

*OR* Odds ratio, *CI* Confidence interval

## Discussion

Overall, this study found that cleaning spare rooms was risk factors for HFRS, while storing food and crops properly at home showed protective effect.

It is possible that hantavirus can be infected by inhaling the aerosol and ingesting the food contaminated by the rodent excreta, that is, the virus may transmit through respiratory and digestive tracts [5, 8, 10–12]. The reason why cleaning spare rooms was the influencing factor might be that the human activities in the spare rooms were infrequent and these rooms were poorly ventilated, which was conducive to rodent reproduction. When people enter these closed rooms to clean, they are likely to be infected by inhaling aerosols containing the virus. Ruan’s [17] and Li’s [18] researches showed that sprinkling during cleaning had a protective effect for the infection. Unfortunately, sprinkling while cleaning spare rooms at home did not enter the equation in this study, the influence of this factor could not be further analyzed.

Studies using multivariate logistic regression analyses [13, 17, 19–22] found that eating the food contaminated by the rats excreta may be one of the risk factors, which was consistent with our finding. Thus, taking proper care of food and crops, just like using lidded buckets or cabinets to store food can effectively avoid contamination by rodent excreta.

This study had several strengths. Firstly, this study was a community-based case-control study, which was less prone to selection bias than hospital-based case -control study, and the neighborhood-matched design made cases and control subjects similar for certain variables. A number of potential confounding factors including social status, economic conditions, health status, housing conditions, etc. were able to be adjusted. Meanwhile, the neighborhood controls were chose in the same community but not adjacent to the cases, ensuring that the factors including career choices, environmental exposure, etc. were not over over-matched. Secondly, investigations were conducted within 1 week after the disease onset, which could reduce the recall bias in reporting the lifestyle characteristics. Last but not least, the investigation lasted over 1 year, covering all the peak incidence of the whole year. More detailed information could be collected compared to some studies [22–24] with shorter investigation time. Lastly, the study was representative of the population in Liangyungang because of the use of community-based study subjects, which made it meaningful for us to carry out suitable prevention and control measures.

This study was also subject to several limitations. Firstly, the sample size was relatively small compared to previous studies [13, 17, 18, 20, 23]. Secondly, the incidence varies significantly in different seasons according to the disease surveillance. This study was only carried out in two epidemic areas of the same city, thus it might not be representative of the general Chinese population.

The results of this study support the view that hantavirus infection is related to the behavioral risk factors. Based on current study, the indoor sanitary condition not only directly affects the reproduction and living conditions of the rodents, but also has important effect on the transmission. Therefore, apart from strengthening the surveillance of rodent epidemics and carrying out rodent control activities vigorously, it may have better prevention effects on HFRS if we improve environmental sanitation conditions and enhance personal protection may have better prevention effects on HFRS.

Unfortunately, among all the recognized risk factors, direct contact with rodents (all home and workplace rodents) did not enter the final regression equation, thus it was impossible to determine its association with HFRS. It might be related to the factors such as the relatively small sample size, the limited selection of the respondents and the investigation area.

## Conclusions

Within the above mentioned limitations, our study further demonstrated that the virus may transmit through respiratory and digestive tracts and proved that preventative measures that reduce exposure to infected animal excreta can prevent the infection. More attention should be paid to promote comprehensive health education and behaviour change among high-risk populations in the HFRS endemic area.

The prevention and control measures should start from three aspects including managing the source of infection, cutting off the transmission route and protecting the susceptible population. Firstly, preventing and exterminating rodents with drugs, machinery and other methods is crucially important in managing the infection source. Also, good personal hygiene and food hygiene remain to be a considerable aspect in cutting of transmission, thus we need to improve the environment condition through preventing rodents’ excreta from polluting food, and not contacting rodents and their excreta by hand. Moreover, emphasis should be put on vaccinating. At present, the inactivated HFRS vaccines developed in China have been used in epidemic areas and have been proved to have good protective effects.

## Data Availability

The datasets used and/or analysed during the current study are available from the corresponding author on reasonable request.
